# Corticomotor Excitability is Increased Following an Acute Bout of Blood Flow Restriction Resistance Exercise

**DOI:** 10.3389/fnhum.2015.00652

**Published:** 2015-12-02

**Authors:** Christopher Roy Brandner, Stuart Anthony Warmington, Dawson John Kidgell

**Affiliations:** ^1^Centre for Physical Activity and Nutrition Research, School of Exercise and Nutrition Sciences, Deakin UniversityMelbourne, Burwood, VIC, Australia; ^2^Talent Identification Unit, Sport Science Department, Aspire AcademyDoha, Qatar; ^3^Department of Rehabilitation, Nutrition and Sport, School of Allied Health, La Trobe UniversityMelbourne, VIC, Australia

**Keywords:** intracortical inhibition, kaatsu, motor cortex plasticity, strength training, transcranial magnetic stimulation, vascular occlusion

## Abstract

We used transcranial magnetic stimulation (TMS) to investigate whether an acute bout of resistance exercise with blood flow restriction (BFR) stimulated changes in corticomotor excitability (motor evoked potential, MEP) and short-interval intracortical inhibition (SICI), and compared the responses to two traditional resistance exercise methods. Ten males completed four unilateral elbow flexion exercise trials in a balanced, randomized crossover design: (1) heavy-load (HL: 80% one-repetition maximum [1-RM]); (2) light-load (LL; 20% 1-RM) and two other light-load trials with BFR applied; (3) continuously at 80% resting systolic blood pressure (BFR-C); or (4) intermittently at 130% resting systolic blood pressure (BFR-I). MEP amplitude and SICI were measured using TMS at baseline, and at four time-points over a 60 min post-exercise period. MEP amplitude increased rapidly (within 5 min post-exercise) for BFR-C and remained elevated for 60 min post-exercise compared with all other trials. MEP amplitudes increased for up to 20 and 40 min for LL and BFR-I, respectively. These findings provide evidence that BFR resistance exercise can modulate corticomotor excitability, possibly due to altered sensory feedback via group III and IV afferents. This response may be an acute indication of neuromuscular adaptations that underpin changes in muscle strength following a BFR resistance training programme.

## Introduction

Blood flow restriction (BFR) in combination with light-load resistance exercise (20–30% 1 repetition maximum [1-RM]) is a novel exercise technique that has been shown to increase muscle activity (as measured by surface and intramuscular electromyography [EMG]) to levels greater than non-BFR light-load resistance exercise (Moritani et al., 1992; Yasuda et al., 2006), while being similar to traditional heavy-load resistance exercise (≥65% 1-RM; Takarada et al., 2000; Yasuda et al., 2008). An increase in muscle activation during BFR resistance exercise indicates a modification in the orderly recruitment of motor units to include fast-twitch muscle fibers despite the use of light-loads (Moritani et al., 1992). The increase in EMG amplitude has been speculated to be related to a number of factors including: (i) pooling of blood distal to the cuff (Iida et al., 2007); (ii) an increase in accumulation of metabolites within muscle such as blood lactate (Yasuda et al., 2014); and (iii) altered availability of metabolic substrates such as oxygen, glucose, and free fatty acids (Moritani et al., 1992; Suga et al., 2012); or a combination of these. In any case, it is most likely that the ischemic/hypoxic environment associated with BFR resistance exercise alters sensory feedback via group III and IV muscle afferents to modify muscle activation (Yasuda et al., 2010).

Since voluntary muscle activity arises from the level of the human primary motor cortex (M1), it is reasonable to hypothesise that during BFR resistance exercise there may be some form of modulation within M1 that alters the pattern of motor unit recruitment (Rothwell, 1994; Nolte, 2002). Currently, information regarding neuromuscular function during BFR resistance exercise or the post-exercise time course response to this type of exercise remains limited (for review, see Karabulut et al., 2007). As discussed, previous studies have reported increases in surface and/or intramuscular EMG (Moritani et al., 1992; Takarada et al., 2000; Yasuda et al., 2006, 2008), while two studies have utilized the twitch interpolation technique to determine muscle activation levels (Moore et al., 2004; Karabulut et al., 2010). Furthermore, using near infrared spectroscopy it was observed that unilateral elbow flexion exercise (20% 1-RM) with BFR (130% systolic blood pressure; 130–170 mmHg) increased cerebral blood flow to a greater extent than a non-BFR control, suggesting that the contralateral M1 was activated to a greater magnitude during an acute resistance exercise bout in combination with BFR (Morita et al., 2010). It is important to note that these are peripheral measures of neuromuscular activity, and not a direct measure of the corticomotor structures involved in modulating voluntary force production. Therefore, a limitation of the current BFR resistance exercise data is that it provides no information regarding the output from the M1.

Corticomotor excitability and inhibition can be examined using transcranial magnetic stimulation (TMS) to measure the change in amplitude of motor evoked potentials (MEPs) recorded via EMG from the target muscle (Terao and Ugawa, 2002). When normalized to a peripheral nerve stimulus, a modification in the amplitude of MEPs following an intervention reflect the excitability of corticomotor and spinal motoneurons (Terao and Ugawa, 2002). While no study has used TMS following BFR resistance exercise to examine corticomotor excitability, there is some evidence to suggest that corticomotor excitability and twitch force are modified following acute bouts of resistance exercise under normoxia (Carroll et al., 2008; Selvanayagam et al., 2011) and systemic hypoxic conditions (Millet et al., 2012). Further evidence of modifications in corticomotor excitability and inhibition has been observed in response to temporary ischemic nerve deafferentation (Brasil-Neto et al., 1993a,b; Ridding and Rothwell, 1995). However, it is important to note that these studies did not use an exercise intervention, and only examined corticomotor excitability and inhibition under resting ischemic conditions. For example, to induce complete ischemic nerve block of the hand muscles (31.7 ± 3.8 min), a tourniquet was applied across the elbow at a pressure of 125–130% resting systolic blood pressure (200–250 mmHg; Ziemann et al., 1998a). MEP amplitudes of muscles proximal to the tourniquet (biceps brachii and deltoid) measured during the late onset of ischemia increased by approximately 30% when normalized to baseline, and were approximately 60% greater at 20 min post-ischemia. Interestingly, MEP amplitude remained elevated for at least 60 min following deflation of the tourniquet (Ziemann et al., 1998a). The increase in MEP amplitude likely reflects a change in the excitability or representation of these muscles at the level of the M1, given that measurement of spinal excitability assessed via transcranial electrical stimulation and Hoffmans reflex remain unchanged (Brasil-Neto et al., 1993b). The observed increase in MEP amplitudes are suggested to be mediated by the strengthening (e.g., long-term potentiation) or weakening (e.g., long-term depression) of pre-existing synaptic connections (Ziemann et al., 1998b), or the removal of local inhibition of corticomotor neurons that are responsible for movement in the target muscle proximal to the external pressure cuff (Ziemann et al., 1998a).

Given that corticomotor inputs are required for motor unit recruitment (Rothwell, 1994; Nolte, 2002), it seems plausible to suggest that during BFR resistance exercise there may be some form of modulation within the M1 that alters the pattern of motor unit recruitment. Therefore, the purpose of this study was to investigate whether an acute bout of BFR resistance exercise of the biceps brachii differentially modulated corticomotor excitability and short-interval intracortical inhibition (SICI). Specifically, we examined whether any changes in corticomotor excitability and SICI were different between BFR resistance exercise and more traditional training methods such as heavy- and light-load resistance exercise. In addition, due to variations in published techniques to apply BFR (e.g., the duration of restriction and applied exercising cuff pressure) that may affect responses to exercise with BFR (Fahs et al., 2012), the present study also compared two common protocols to conduct BFR resistance exercise (continuous and intermittent BFR application). It was hypothesized that an acute bout of BFR resistance exercise of the biceps brachii would rapidly modulate elements of corticomotor plasticity, as reflected by changes in corticomotor excitability and SICI.

## Materials and Methods

### Participants

Ten (*n* = 10) male participants (22 ± 2 years; 178.5 ± 6.8 cm; 71.6 ± 6.3 kg) volunteered to take part in the study, and provided written informed consent to the experimental procedures prior to participation. Participants had no known history of peripheral or neurological impairment, cardiovascular, pulmonary, or metabolic disease, musculoskeletal injuries, or self-reported smoking. Additionally, none of the participants had involvement in any kind of resistance training in the previous 6 months. This study was approved by the Human Research Ethics Committee of Deakin University, and all experiments were conducted according to the standards established by the Declaration of Helsinki.

### Experimental Design

**Figure 1:**
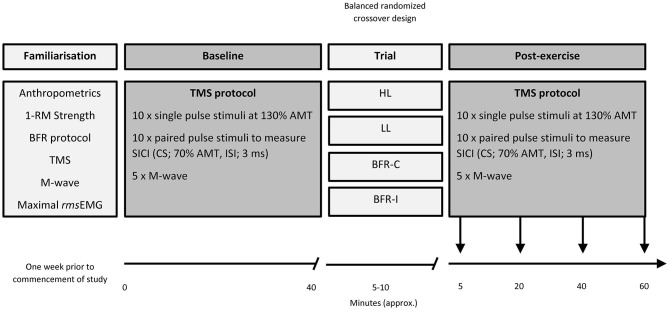
**Organization of the study.** 1-RM, one repetition maximum; AMT, active motor threshold; BFR-C, continuous blood flow restriction (BFR); BFR-I, intermittent blood flow restriction; CS, conditioning stimulus; HL, heavy-load resistance exercise; ISI, interstimulus interval; LL, light-load resistance exercise; *rms*EMG, root mean square of the electromyography.

For a graphical representation of the organization of the study, the reader is directed to Figure 1. Prior to beginning the study, participants underwent a familiarization session that involved: (i) anthropometric measurements and (ii) strength testing to evaluate maximal voluntary dynamic elbow flexor muscle strength (1-RM). Following this visit, in a balanced randomized crossover design, participants attended the laboratory on four separate occasions, each separated by at least 7 days. At each visit, participants completed one of four resistance exercise trials. The four trials were heavy-load resistance exercise (HL), light-load resistance exercise (LL), and two BFR trials; one where the pressure was applied continuously throughout the duration of the exercise bout including rest periods (BFR-C) and the other whereby the pressure was applied intermittently during exercise only (BFR-I). Participants were instructed to avoid caffeine, medications, and exercise on the day of testing. All four visits to the laboratory were at the same time of day. Prior to beginning each trial (Baseline), MEPs were recorded for single- and paired-pulse TMS and was applied to the M1 contralateral to the exercised biceps brachii. Single- and paired-pulse TMS was conducted again four times post-exercise. The first set of data was collected 5 min post-exercise to avoid the period when muscle effects are largest (i.e., post-exercise MEP depression/facilitation; Brasil-Neto et al., 1993a), then again at 20, 40 and at 60 min post-exercise. Direct muscle responses (M_MAX_) were obtained from the biceps brachii by supramaximal electrical stimulation of the brachial plexus (Erb’s point) under resting conditions prior to TMS at all-time points. All data (single- and paired-pulse TMS, as well as M_MAX_) were collected with the participant in a seated position, while the resistance exercise trials were performed with the participant standing.

### Maximal Strength Testing

Participants performed a standard unilateral elbow flexion 1-RM test following a previously verified protocol (Brandner et al., 2015). The initial starting weight was chosen based on the participants’ estimation of strength. Participants performed the 1-RM test standing, holding a weighted dumbbell with their dominant hand, with their elbow in full extension, forearm supinated, and the opposite arm placed behind their back while standing against a wall to prevent excessive body movement. Participants were then asked to flex their arm and lift the dumbbell as if doing a standard “bicep curl”. If the trial was successful, the weight of the dumbbell was increased in increments of 0.5 kg (or greater if appropriate) on each trial after a 3 min recovery period to minimize the development of muscular fatigue. This procedure continued until the participant could no longer perform one full repetition, and the prior trial served as their 1-RM strength. In addition, in order to quantify the appropriate level of muscle contraction during TMS testing, participants completed a maximal voluntary isometric contraction (MVIC) of the dominant biceps brachii. Participants stood in the anatomical position, with the hand supinated and maintaining 90 degrees of elbow flexion. The researcher placed an adjustable weighted dumbbell in the palm of their hand. Participants were instructed to grasp the dumbbell and maintain 90 degrees of elbow flexion for 3 s, without movement of the abdomen or altering their posture. The maximal load that could be held static with correct technique served as their MVIC. Maximal root mean squared electromyography (*rms*EMG) for the bicep was obtained during the 3 s hold of their MVIC.

### Resistance Exercise Procedure

In all trials participants performed supervised unilateral elbow flexion/extension exercise (i.e., a standard series of dumbbell bicep curls) to a repetition timing monitored by a metronome (2 s concentric; 2 s eccentric). This method has been shown to enhance the demand on the nervous system (Ackerley et al., 2007) and has been used previously (Brandner et al., 2015). Table 1 displays the sets/reps regimen for all trials in accordance with standard protocols to conduct each type of exercise (Fahs et al., 2012; Brandner et al., 2015). For HL, participants completed four sets (6–8 repetitions; 80% 1-RM) with 2.5 min rest between sets. For LL, BFR-C and BFR-I, participants completed one set of 30 repetitions followed by three sets of 15 repetitions (20% 1-RM) with 30 s rest between sets. The total time to complete HL was 9 min, whereas all other trials were 6.5 min.

**Table 1:** **Maximum strength (1-RM) and exercise workload characteristics for each trial**.

TRIAL	1-RM (%)	Load (kg)	Sets (Reps)	Restriction pressure	
				(%SBP)	(mmHg)
1-RM		17.5 ± 1.2
HL	80	14.1 ± 1.0	4 (6–8)
LL	20	3.5 ± 1.0	4 (30, 15, 15, 15)
BFR-C	20	3.5 ± 1.0	4 (30, 15, 15, 15)	80	94 ± 4
BFR-I	20	3.5 ± 1.0	4 (30, 15, 15, 15)	130	153 ± 5

*1-RM, one repetition maximum; BFR-C, continuous blood flow restriction; BFR-I, intermittent blood flow restriction; HL, heavy-load resistance exercise; LL, light-load resistance exercise; SBP, systolic blood pressure. Data reported as Mean ± SEM*.

### Blood Flow Restriction Protocol

For both BFR trials (BFR-C and BFR-I), participants wore a pneumatic cuff (52 cm long, 10.5 cm wide; bladder length 45 cm, bladder width 8 cm) around the most proximal portion of the arm, connected to an automatic tourniquet system (A.T.S. 3000, Zimmer Inc., OH, USA). With the participant standing, cuff pressure was set to 50 mmHg for 30 s, then released for 10 s. This cycle was repeated with an additional 20 mmHg on each inflation until reaching the final exercise pressure for BFR-C (80% resting systolic blood pressure; 94 ± 4 mmHg) and BFR-I (130% systolic blood pressure; 153 ± 5 mmHg). For BFR-I only, the cuff was completely deflated (i.e., 0 mmHg) during the rest periods between sets. This deflation was performed to improve participant comfort and tolerance, and is a method of BFR application used previously (Suga et al., 2012; Brandner et al., 2015).

### Electromyography and Transcranial Magnetic Stimulation

Surface EMG was recorded from the biceps brachii muscle of the exercised (dominant) arm, using 9 mm cup electrodes (Electrode model: MLAWBT9, ADInstruments, Bella Vista, Australia). Two electrodes were placed over the muscle belly of the biceps brachii, and one reference electrode was positioned on the participants’ hand. The participants’ skin was shaved and swabbed with 70% isopropyl alcohol prior to electrode placement to ensure a clear signal was obtained. Surface EMG signals were amplified (×1000), bandpass filtered (high pass at 13 Hz, low pass at 1000 Hz), digitized online at 2 kHz for 500 ms, recorded and analyzed using PowerLab 4/35 (ADInstruments, Bella Vista, Australia). Single- and paired-pulse TMS of the M1 was applied using two Magstim 200^2^ stimulators (Magstim Co Ltd., UK) to produce active MEPs in the biceps brachii with a 70 mm figure eight coil (external loop diameter of 9 cm). The handle of the TMS coil was positioned over the “optimal” site (the location on the M1 that evokes the maximum MEP amplitude to the muscle of interest), and oriented so that the axis of the intersection between the two loops was oriented at approximately 45 degrees to the sagittal plane. It was anticipated that this arrangement induced a posterior-anterior current flow across the motor strip for activating the dominant M1 and right biceps brachii muscle (Kidgell et al., 2010). To ensure consistency of coil placement throughout testing, participants wore a snug fitting cap, positioned with reference to the nasion-inion and interaural lines. The cap was marked with 1 cm spaced sites in a latitude-longitude matrix to ensure consistent coil position throughout the testing protocol and for repeated testing sessions over the period of the study. The cap and coil position was checked regularly to ensure the positioning of the TMS coil was consistent. All stimuli were delivered during a low level isometric contraction of the biceps brachii, which were performed by supinating the hand and maintaining 90 degrees of elbow flexion. During all subsequent TMS testing, holding the arm in this joint position without resistance equated to 3.93 ± 0.40% of the maximal *rms*EMG, with consistent low level muscle activation confirmed by recording pre-stimulus *rms*EMG for the 100 ms epoch prior to the delivery of each stimuli. Active motor threshold (AMT) was established as the stimulus intensity at which a small MEP (200 μV in three out of five consecutive trials) during a low level isometric contraction of the biceps brachii at 3.93 ± 0.40% maximal *rms*EMG activity (Wilson et al., 1993). The stimulus intensity started at 50% maximal stimulator output (MSO) and was altered in increments of ±1% of MSO until the appropriate threshold level was achieved.

### Motor Evoked Potentials and Short-Interval Intracortical Inhibition

The single-pulse TMS protocol to measure MEP amplitude comprised 10 unconditioned stimuli elicited at a stimulus intensity of 130% AMT. This was followed by 10 paired-pulse TMS stimuli to induce SICI. The paired-pulse stimuli comprised an initial subthreshold conditioning stimulus at 70% AMT, followed by a suprathreshold test stimulus at 130% AMT. The interstimulus interval was 3 ms. For each trial, AMT was determined at baseline and each subsequent time point. Therefore, at each time point, in each trial, the test stimulus for both single- and paired-pulse TMS was always equivalent, and was always 130% AMT, while the paired-pulse conditioning stimulus was always 70% AMT. For both single- and paired-pulse TMS, the 10 protocols were delivered at random intervals every 5 to 12 s to avoid stimulus anticipation, and 60 s rest was provided between the single- and paired-pulse phases to reduce the possibility of muscle fatigue.

### Maximal Compound Muscle Action Potential

Direct muscle responses were obtained from the right biceps brachii by supramaximal percutaneous electrical stimulation of the brachial plexus (Erbs point) under resting conditions at all time-points. A Digitimer (DS7A, Hertfordshire, UK) constant-current electrical stimulator (pulse duration 1 ms) was used to deliver each electrical pulse via positioning bipolar electrodes in the supraclavicular fossa. The stimuli were delivered while the participant sat in an upright position, with the arm resting comfortably in the lap, producing no detectible background EMG. An increase in current strength was applied to the brachial plexus until there was no further increase in the amplitude of surface EMG response (M_MAX_). To ensure maximal responses, the current was increased an additional 20% and the average M_MAX_ was obtained from five stimuli, with a period of 5–10 s separating each stimulus.

### Data Analyses

Pre-stimulus *rms*EMG activity was determined in the biceps brachii 100 ms prior to each TMS stimulus during each condition. Any pre-stimulus *rms*EMG that exceeded 5 ± 3% of maximal *rms*EMG were discarded and the trial repeated. The peak-to-peak amplitude of MEPs evoked as a result of stimulation was measured in the biceps brachii muscle contralateral to the cortex being stimulated in the period 10–50 ms after stimulation. MEP amplitudes were analyzed using LabChart software (8, ADInstruments, Bella Vista, Australia) after each stimulus was automatically flagged with a cursor, providing peak-to-peak values in μV and were then normalized to M_MAX_. Average MEP amplitudes were obtained separately for single- and paired-pulse TMS for each stimulation block (20 trials for each time point). SICI was calculated using the following equation: (1 – PP/SP) × 100. This calculation, adapted from Lackmy and Marchand-Pauvert (2010), has a direct relationship with SICI (unlike the traditional method for calculating SICI ratio). For example, a decrease in inhibition following the intervention would be depicted by a decrease in the numerical value.

### Statistical Analysis

All data were screened for normality using Shapiro-Wilk and Kolmogorov-Smirnov tests, and were found to be normally distributed. Consequently, a repeated measures ANOVA for within factors of trial (HL, LL, BFR-C and BFR-I) and time (Baseline, 5, 20, 40 and 60 min post) was used to examine the trial and time effects on *rms*EMG, AMT, MEP amplitude, SICI, and M_MAX_. When appropriate, *post hoc* (Tukey) analyses for pairwise comparisons of means were used when significant interactions were found. For all tests, the Huynh-Feldt correction was applied if the assumption of sphericity was violated. Alpha was set at *p* ≤ 0.05, and all results are displayed as mean ± standard error of the mean (SEM) unless stated otherwise.

## Results

### Baseline Characteristics and Strength

Mean elbow flexor strength is displayed in Table 1, along with the average exercising load for each trial.

There were no differences in AMT, M_MAX_, MEP amplitude, SICI, and *rms*EMG between trials at baseline (all *p* ≥ 0.05). In addition, the mean TMS stimulator output for AMT, single-pulse, paired-pulse and M_MAX_ were not different between trials or time points, therefore these were averaged across all trials and are presented in Table 2.

**Table 2:** **Baseline corticomotor responses and TMS variables**.

TMS variables	
M_MAX_ (mV)	11.92 ± 1.52
Stimulator output for M_MAX_ (mA)	62.50 ± 13.50
AMT (mV)	0.33 ± 0.10
AMT (% MSO)	39.4 ± 2.0
Unconditioned (single-pulse; % MSO)	51.2 ± 2.7
Conditioning (paired-pulse; % MSO)	27.5 ± 1.5
Maximal *rms*EMG (mV)	1.62 ± 0.20

*AMT, active motor threshold; Maximal *rms*EMG, maximal root mean squared electromyography; *M*_MAX_, maximal compound peripheral muscle action potential, MSO; maximal stimulator output. Data reported as Mean ± SEM*.

### Pre-Stimulus *rms*EMG and M_MAX_

**Figure 2:**
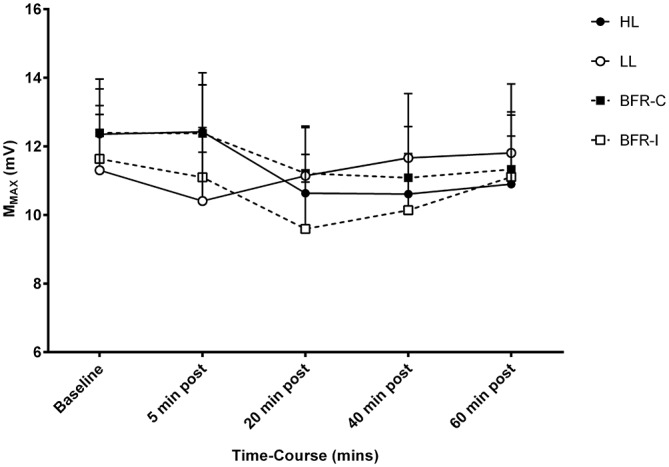
**M_MAX_ amplitude following resistance exercise.** HL, heavy-load resistance exercise; LL, light-load resistance exercise; BFR-C, blood flow restriction with continuous inflation of cuff pressure; BFR-I, blood flow restriction with intermittent inflation of cuff pressure.

The averaged pre-stimulus *rms*EMG values recorded were not different between groups at baseline. In addition, there were no significant differences between trials or time-points, and therefore no time-by-trial interactions were detected for *rms*EMG for the 100 ms prior to stimulation (all *p* ≥ 0.05). Similarly, for M_MAX_, there were no time-by-trial interactions, main effects for time or trial detected (all *p* ≥ 0.05; Figure 2).

### Active Motor Threshold and Corticomotor Excitability

TMS stimulus output required to evoke AMT for biceps brachii was not different between trials (Table 2). Similarly, AMT was not different between trials at baseline. Therefore, AMT was averaged across all trials and is displayed in Table 2.

**Figure 3:**
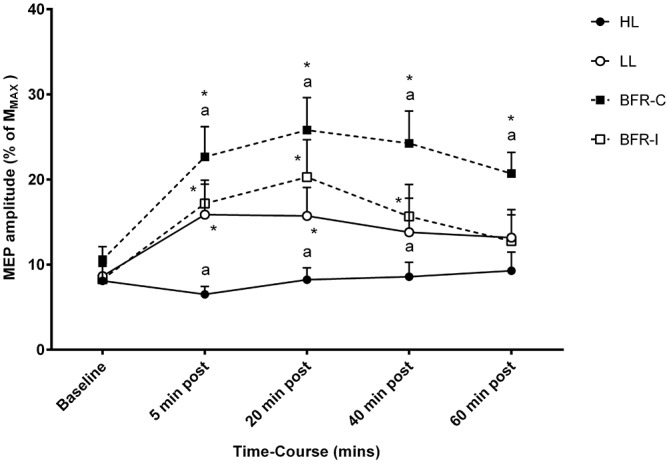
**MEP amplitude relative to M_MAX_ following resistance exercise.** HL, heavy-load resistance exercise; LL, light-load resistance exercise; BFR-C, blood flow restriction with continuous inflation of cuff pressure; BFR-I, blood flow restriction with intermittent inflation of cuff pressure. *Indicates significantly different to Baseline (*p* < 0.05); “a” indicates significantly different to all others trials (*p* < 0.05).

Overall, a significant time-by-trial interaction was detected for corticomotor excitability (Figure 3; *F*_(12,108)_ = 4.223; *p* ≤ 0.001). Univariate *post hoc* analyses revealed a significant increase in MEP amplitude at 5 min post-exercise following LL (*p* ≤ 0.001), BFR-I (*p* ≤ 0.001), and BFR-C (*p* ≤ 0.001) relative to HL. In addition, MEP amplitude was significantly greater following BFR-C compared with LL (*p* ≤ 0.01) and BFR-I (*p* ≤ 0.05). MEP amplitude increased rapidly at 5 min post compared with baseline following all trials except for HL (*p* ≤ 0.001). At 20 min post-exercise, the magnitude of the increase in MEP amplitude remained significant for LL (*p* ≤ 0.01), BFR-I (*p* ≤ 0.001), and BFR-C (*p* ≤ 0.001) compared with HL. Furthermore, MEP amplitude remained significantly elevated following BFR-C compared with LL (*p* ≤ 0.001) and BFR-I (≤ 0.05). Relative to baseline, MEP amplitude remained significantly greater 20 min post-exercise following all trials (*p* ≤ 0.01) except HL. Similarly, at 40 min post-exercise, MEP amplitude remained significant for BFR-C compared with HL (*p* ≤ 0.001), LL (*p* ≤ 0.001), and BFR-I (*p* ≤ 0.001). In addition, MEP amplitude was greater for LL and BFR-I compared with HL (*p* ≤ 0.05). Relative to baseline, MEP amplitude remained elevated following both BFR-I (*p* ≤ 0.01) and BFR-C (*p* ≤ 0.001) only. Interestingly, at 60 min post-exercise, MEP amplitude was still significantly elevated following BFR-C relative to HL (*p* ≤ 0.001), LL (*p* ≤ 0.01) and BFR-I (*p* ≤ 0.001), but there were no differences between any other trials. MEP amplitude remained significantly elevated above baseline for BFR-C only (*p* ≤ 0.001).

### Short Interval Intracortical Inhibition

**Figure 4:**
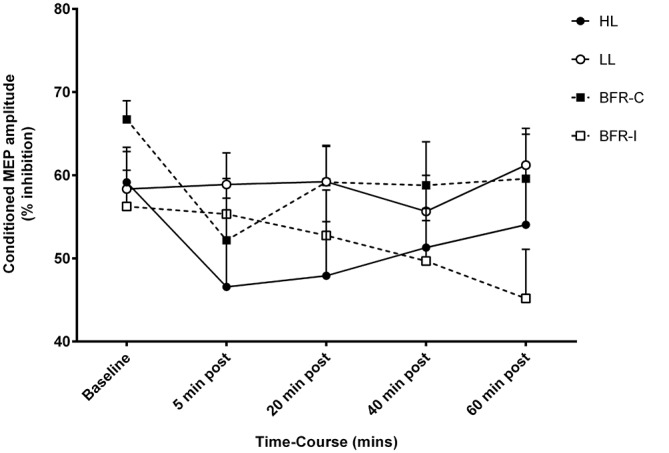
**Short-interval intracortical inhibition (SICI) amplitude following resistance exercise.** HL, heavy-load resistance exercise; LL, light-load resistance exercise; BFR-C, blood flow restriction with continuous inflation of cuff pressure; BFR-I, blood flow restriction with intermittent inflation of cuff pressure.

The conditioning stimulus intensity required to evoke SICI for biceps brachii was not different between trials (Table 2). The conditioning stimulus intensity required to evoke SICI for biceps brachii was not different between trials (Table 2). There were no time-by-trial interactions (Figure 4; *F*_(12,108)_ = 1.485; *p* = 0.141), main effects for time (*F*_(4,36)_ = 2.518; *p* = 0.058) or trial (*F*_(3,27)_ = 1.182; *p* = 0.335).

## Discussion

The main findings of the present study were: (i) overall, the increase in MEP amplitude of the biceps brachii was greater following BFR-C compared with all other trials, and remained so for up to 60 min post-exercise; (ii) both BFR trials rapidly increased corticomotor excitability; (iii) MEP amplitude was unaffected by traditional heavy-load resistance exercise; and (iv) no modifications were detected for SICI post-exercise following all trials. These results support our hypothesis, and suggest that in order to induce rapid and long-lasting increases in corticomotor excitability during BFR resistance exercise of the biceps brachii, continuous low-pressure application is preferential to intermittent high-pressure application.

Currently, there is limited data available that has assessed neuromuscular function in response to BFR resistance exercise. Several studies have reported acute changes in peripheral measures of the neuromuscular system, such as increases in surface EMG during BFR resistance exercise (Moritani et al., 1992; Takarada et al., 2000; Yasuda et al., 2006, 2008), or utilizing the twitch interpolation technique to determine changes in muscle activation levels following training (Moore et al., 2004; Karabulut et al., 2010). However, the present study is the first to directly measure the potential cortical structures involved in modulating corticomotor excitability and inhibition following BFR resistance exercise. It was observed that MEP amplitude increased rapidly (at 5 min post-exercise) following BFR resistance exercise regardless of the pattern/timing of restriction and final inflation pressure. However, MEP amplitude was facilitated for 60 min following BFR-C, but returned to baseline by 40 min post-exercise for BFR-I. These results suggest that the pattern/timing of cuff restriction application during BFR resistance exercise is an important factor in modulating corticomotor excitability. One potential limitation to the current study was that we did not include a BFR only (no exercise) trial. Nevertheless, Ziemann et al. (1998a) has previously examined similar time-course corticomotor responses during and following ischemic nerve deafferentation, and showed that while corticomotor excitability was increased during the late stage of ischemia (approximately 5 min after nerve block was achieved at 31.7 ± 3.8 mins), and for 60 min post-ischemia, the increase from baseline was only significant at 20 min post-ischemia (~60% increase). Reduced oxygen availability has been suggested to be a potential mechanism behind the increased EMG activity seen during BFR resistance exercise (Yasuda et al., 2010). However, under systemic hypoxic conditions, MEP amplitude has been shown not to increase at rest within 60 min of exposure (Rupp et al., 2012; Goodall et al., 2014). In addition, no effect for SICI has been found at rest under systemic hypoxic conditions (Rupp et al., 2012), or during and following ischemic nerve deafferentation (Ziemann et al., 1998a). Given that the duration of BFR in the current study was less than 10 min, and restriction of blood flow for this duration without exercise is not known to induce muscular adaptations, it is not expected that any changes in corticomotor excitability or inhibition would occur in a BFR only control trial of this short duration. Therefore, we are confident that our results are likely to be as a result of the combination of BFR and light-load resistance exercise. Based on this finding, we propose that when elbow flexion exercise is performed with 20% 1-RM, the application of low-pressure continuous BFR should be used in order to induce the greatest modification in corticomotor excitability. Future studies should examine if similar responses would be observed during muscular contractions at higher intensities, or for other resistance exercises (e.g., for the lower-body).

Of particular interest to this study was the effect of BFR resistance exercise on modulating corticomotor excitability and inhibition in comparison with more traditional resistance exercise techniques. We found no change in corticomotor excitability following the HL trial, which was somewhat unexpected. MEP amplitude has been observed to increase during sustained isometric contractions of the elbow flexors (Sacco et al., 1997), as well as following acute bouts of ballistic resistance exercise of small hand muscles (Carroll et al., 2008; Selvanayagam et al., 2011). In contrast, and in agreement with results from the current study, no change in MEP amplitude was reported following exercise of the hand muscles at 80% MVC (Hortobagyi et al., 2011), or following five sets of 6–10 repetitions of the elbow flexors (load not reported; Jensen et al., 2005). It is possible that MEP amplitude did not change following the HL trial due to central fatigue mechanisms (Gandevia, 2001). While maximal force wasn’t measured post-exercise in order to determine the level of muscular fatigue, because no change in SICI or M_MAX_ was observed, we hypothesize that MEP amplitude wasn’t modified in the current study (and others e.g., Jensen et al., 2005), due to the limited centrally challenging nature of the HL trial. For example, while the heavy-loads utilized could be considered challenging to the neuromuscular system, the addition of completing each repetition to external pacing (i.e., with a metronome) has been shown to increase the complexity of the movement resulting in increased MEP amplitude following motor skill learning tasks (Jensen et al., 2005) and short-term resistance training programmes (Kidgell et al., 2010; Weier et al., 2012). However, in the present study some participants were unable to keep time with the required contraction rate (2 s concentric, 2 s eccentric) due to the heavy-load. As such, this may have limited the centrally challenging nature of the HL trial, and so may explain why we did not observe any acute modulation in MEP amplitude. In contrast, in the present study during the LL trial using the same external pacing as HL, we observed a rapid increase in MEP amplitude that remained elevated 20 min post-exercise. This data suggests that either the load used during LL didn’t induce fatigue thus no depression in post-exercise MEP amplitude, or that the combination of exercise to external pacing and a higher number of repetitions was responsible for the increase in MEP amplitude. Moreover, using the same light-load resistance exercise and repetition timing but with an applied BFR, we observed an even larger increase in MEP amplitude, and a longer lasting facilitation of MEP amplitude post-exercise when compared with both HL and LL trials. Therefore, the net increase in corticomotor excitability seen in the present study not only provides support for benefits of BFR resistance exercise in healthy populations, but may also be important for clinical populations that require increased motor function such as the elderly, stroke patients, and following musculoskeletal injury.

It is well documented that large motor units (and their associated fast-twitch muscle fibers) are preferentially recruited during BFR resistance exercise with light-loads (Moritani et al., 1992; Takarada et al., 2000; Yasuda et al., 2006, 2010; Karabulut et al., 2007). This increase in muscle activation during BFR resistance exercise is similar to heavy-load resistance exercise, and greater than light-load resistance exercise without BFR (Moritani et al., 1992; Takarada et al., 2000; Yasuda et al., 2006). It has been proposed that the high levels of external compression, reduced blood flow, and an ischemic/hypoxic intramuscular environment may all play a role in stimulating the increase in muscle activation via group III and IV muscle afferents (Moritani et al., 1992; Karabulut et al., 2007; Yasuda et al., 2010). Given that sensory feedback to cortical and/or subcortical areas during exercise and under ischemic/hypoxic conditions has been proposed to alter muscle activation and corticomotor excitability (Gandevia et al., 1996; Christie and Kamen, 2014), evidence from the present study further supports a potential role of group III and IV muscle afferents in modulating corticomotor excitability with BFR. While there is also evidence to suggest that sensory feedback from group III and IV muscle afferents plays a role in altering cortical inhibition (Christie and Kamen, 2014), SICI remained unchanged in the present study following all trials. Previous investigations of SICI on corticomotor plasticity and performance have produced varying results. Ischemia alone has been shown to produce non-significant reductions in SICI (Ziemann et al., 1998a), while SICI has also been shown to decrease following resistance exercise and other motor tasks with increasing levels of force (Rantalainen et al., 2013). In contrast, several studies show SICI to be unchanged as a result of motor skill practice (Rosenkranz and Rothwell, 2006; Schmidt et al., 2011), which supports the findings of the present study in all trials. This suggests that M1 inhibition may not be a primary factor involved in the use dependant modification observed in the M1 following BFR resistance exercise, but seems more likely a result of intracortical facilitation.

The resultant increase in MEP amplitude observed following BFR-C suggests a hyperexcitability of excitatory corticospinal circuits, which may lead to long-lasting adaptations if the intervention is repeated during a training programme, similar to those observed following heavy-load resistance training (Kidgell et al., 2010; Weier et al., 2012). However, it is not known whether the increased corticomotor excitability has any functional outcome such as an increase in muscular strength. Therefore, future studies investigating the neuromuscular adaptations following BFR resistance training using TMS should do so over short- and long-term training durations. We postulate that the increase in MEP amplitude of the biceps brachii in the present study was caused by changes in synaptic efficacy and/or transmission along the corticospinal pathway following BFR resistance exercise. The rapid and long-lasting modulation of MEP amplitude potentially reflects a change in the excitability or representation of the biceps brachii at the level of the M1 because we observed no change in M_MAX_, which indicates that peripheral mechanisms were not responsible for this modification. Furthermore, evidence from ischemic nerve deafferentation shows no change in spinal excitability using transcranial electrical stimulation or Hoffman reflex (Brasil-Neto et al., 1993b; Ridding and Rothwell, 1995). Although our results support this work, we accept that changes in MEP amplitude and SICI are not only affected by the excitability of the M1, but also the excitability of motoneurons at the level of the spinal cord (Classen et al., 1998; Carroll et al., 2011). Therefore, a limitation of the current study was that because we did not obtain any measurements at the level of the spinal cord, it is therefore possible that changes here may also have contributed to the observed alterations in MEP amplitude. Another limitation of the current study was that active MEPs were collected during a low level contraction (3.93 ± 0.40% of maximal *rms*EMG) and were normalized to M_MAX_ values that were collected during muscle relaxation. This variation in muscle activation may prevent the accurate determination of corticomotor excitability and SICI, and should be considered in future studies. Finally, while our method for measuring SICI has been used previously to measure changes in intracortical inhibition following resistance training and motor control tasks (Rantalainen et al., 2013), due to the selected timing and number of time points for measurement post-exercise within the current study design, it was not possible to examine SICI in response to a range of test stimulus intensities. While such an approach may potentially skew our measures of SICI, we think this is unlikely.

## Conclusion

This is the first study to examine corticomotor excitability and inhibition following an acute bout of BFR resistance exercise. It was demonstrated that corticomotor excitability increased rapidly following BFR resistance exercise and remained facilitated for up to 60 min. Interestingly, we found the increase in corticomotor excitability to be greatest following a continuous BFR protocol in comparison with an intermittent BFR protocol and traditional resistance exercise techniques. It is likely that the change in corticomotor excitability was mediated by altered sensory feedback to cortical and/or subcortical areas via group III and IV afferent fibers. This effect may contribute to similar longer-term cortical adaptations that are observed following chronic resistance training with heavy-loads; however this remains to be elucidated. Therefore, future investigations are needed to clarify the impact of these corticomotor adaptations on muscle strength and overall function following BFR resistance training in order to better understand the neuromuscular adaptations that occur following training.

## Author Contributions

Conceived and designed the experiments: CRB, DJK, SAW. Performed the experiments: CRB. Data analysis: CRB, DJK, SAW. Wrote the paper: CRB, DJK, SAW.

## Funding

This study was supported with funds made available via the School of Exercise and Nutrition Sciences, and the Centre for Physical Activity and Nutrition Research, Deakin University, Australia.

## Conflict of Interest Statement

The authors declare that the research was conducted in the absence of any commercial or financial relationships that could be construed as a potential conflict of interest.
